# Multi-Class Weed Recognition Using Hybrid CNN-SVM Classifier

**DOI:** 10.3390/s23167153

**Published:** 2023-08-13

**Authors:** Yanjuan Wu, Yuzhe He, Yunliang Wang

**Affiliations:** Tianjin Key Laboratory of Control Theory & Applications in Complicated Systems, Tianjin University of Technology, Tianjin 300384, China; hyz417871079@stud.tjut.edu.cn (Y.H.); wangyl@tjut.edu.cn (Y.W.)

**Keywords:** weed recognition, precision agriculture, convolutional neural network

## Abstract

The Convolutional Neural Network (CNN) is one of the widely used deep learning models that offers the chance to boost farming productivity through autonomous inference of field conditions. In this paper, CNN is connected to a Support Vector Machine (SVM) to form a new model CNN-SVM; the CNN models chosen are ResNet-50 and VGG16 and the CNN-SVM models formed are ResNet-50-SVM and VGG16-SVM. The method consists of two parts: ResNet-50 and VGG16 for feature extraction and SVM for classification. This paper uses the public multi-class weeds dataset DeepWeeds for training and testing. The proposed ResNet-50-SVM and VGG16-SVM approaches achieved 97.6% and 95.9% recognition accuracies on the DeepWeeds dataset, respectively. The state-of-the-art networks (VGG16, ResNet-50, GoogLeNet, Densenet-121, and PSO-CNN) with the same dataset are accurate at 93.2%, 96.1%, 93.6%, 94.3%, and 96.9%, respectively. In comparison, the accuracy of the proposed methods has been improved by 1.5% and 2.7%, respectively. The proposed ResNet-50-SVM and the VGG16-SVM weed classification approaches are effective and can achieve high recognition accuracy.

## 1. Introduction

Weed damage to crops is serious, and the seedling stage of a crop is the most important time to control weeds. Weed management is needed for optimal yields in crops. At present, the most common form of weed control is widespread pesticide spraying with the advantages of low cost and good weed control, but large amounts of pesticides can affect crops and contaminate the soil. Although precision agriculture is very popular nowadays [1], pesticides applied to the soil can cause contamination because the accuracy cannot reach 100% [2]. Determining how to achieve the accurate and rapid identification of crops and weeds is key to solving this problem. Therefore, it is crucial to research the quick and accurate categorization of crops and weeds and provide technical support for the creation of further mechanical weeders and weeders that precisely apply pesticides [3].

During the current survey, the identification of crops and weeds was based mostly on machine vision, and scholars have distinguished between crop and weed images mainly based on their different characteristics, and the most critical step is the extraction of features. The extracted image features mainly include color [4,5], texture [6], shape [7], edge [8,9], hyperspectral [10,11], etc. These kinds of technology enable the accurate identification of weeds and crops. A large number of experiments have shown that most crops and weeds are different in color, shape, texture, edge, hyperspectral, and other characteristics, which can be used to distinguish between crops and weeds, and then remove the weeds and keep the crops.

Previous authors [12] presented a technique that converted the RGB image of the crop into HSV color space and selected the HSV range of the crop for the corresponding weather conditions based on the histogram of the Hue, Saturation, and Luminosity values of the images, and obtained a binarized image of the original image based on this range, which contained only the crop and not the weeds. The authors [13] converted the RGB image to a Grayscale image and then calculated its GLCM (Gray-level Co-occurrence Matrix) and extracted features according to GLCM. The literature [14] compared and analyzed six features to obtain the optimal feature fusion strategy. To locate and identify weeds and maize seedlings, as well as to lessen the harm weeds do to the growth of maize, the optimal multi-feature fusion in combination with SVM was applied. The authors of [15] used Unmanned Aerial Vehicles (UAVs) to collect images from a chili field in Australia and used several machine learning algorithms such as Random Forest (RF), SVM, and K-nearest Neighbors (KNN) to detect weeds in the images. The authors of [16] developed a robotic vision-based weed knife control system with novel 3D geometric detection algorithms for the automatic weed control of tomato and lettuce crops signals from shaded crop plants while traveling at speeds of up to $3.2\;\mathrm{km}\;\cdot {\;h}^{-1}$ and crop detection accuracy was 97.8% with a detection time of $3.2\;\mathrm{ms}\;\cdot {\;f}^{-1}$. In another article, in order to distinguish between crops and in-row weeds in complicated natural environments, the authors [17] introduced a novel method for processing crop signals in real time using machine vision, where the signal components were machine-readable and aided in the creation of visual characteristics. The proposed crop and weed algorithms were created for robots that used vision to weed. According to research observations, the new approach had a 99.75% accuracy rate for crop recognition and a 98.11% detection rate for sprayable weeds. 

The above-proposed image processing method, although effective in recognizing crops and weeds, requires manual labor and is time-consuming. Weeds in the field are varied, have complex backgrounds, and are affected by many additional factors, such as light, which can cause the pixel values of similar weed images to vary greatly, such that we are unable to recognize objects in an image by explicitly specifying certain features. In addition, image processing methods with low accuracy cannot meet the needs of practical applications.

The remainder of the research is organized as follows. Section 2 examines the related work from recent deep learning-based recognition methods. Section 3 presents the structural illustration and schematic of the algorithm. Section 4 presents the parameters required to train the network, the structural diagram of the proposed approaches, and the evaluation of the proposed approaches. In Section 5, experimental results have been presented and present models are contrasted. The Discussion and Conclusion are provided in Section 6 and Section 7, respectively.

## 2. Related Works

### 2.1. Feature-Based Approaches

Early methods for weed and crop recognition depend primarily on the features created by humans, such as color, texture, and shape.

Using soybean seedlings and related weeds as test subjects, a previous study in the literature [5] developed a weed detection algorithm based on K-means feature learning combined with the convolutional neural network. The model ultimately achieved an accuracy of 92.89%, 1.82% higher than that of a convolutional neural network. The literature [9] described a method for smart farming that uses near-infrared (NIR) and RGB data collected by a UAV to identify crops and weeds. The literature [18] used an edge-detection approach to identify weeds in RGB images of lawns, resulting in accuracies of 67 to 83%. Weed detection systems relying on hyperspectral imaging produced classification accuracies of 88 to 95% [19]. The literature [20] proposed a different kind of remote platform to the traditional methods by using the UAV to provide ultra-high spatial resolution as an alternative and proposed a method for crop row detection, separating crops, weeds, and soil according to different pixels. A K-means classifier was used to perform the classification and decision.

### 2.2. Deep Learning-Based Approaches

Objects in a picture or complete images can be classified using convolutional neural networks (CNNs), a new processing method [21]. Due to the effects of light intensity or soil background, it is more difficult to address these effects with traditional human process features. In comparison, the powerful CNNs extract weed features more consistently and independently of these factors [22]. 

To solve the problem of heavily obscured crop leaves, another study in the literature [23] collected 17,000 images of winter wheat fields to train and validate a CNN model, with a recall of 46.3% and an accuracy of 86.65%. Another study [24] collected 17,509 images of different weeds in Australia and classified these images into a dataset named DeepWeeds, with which two CNN models, GoogLeNet-based Inception-v3 and ResNet-50, were trained and validated with accuracies of 95.1% and 95.7%. Other authors [25] trained CNNs using an image dataset self-built with the aim of distinguishing grasses from broad-leaved classes. It was also compared with SVM, AdaBoost, and RF. The final accuracy of the CNN obtained was over 98%. Previous authors [26] used their improved VGG-16 neural network to estimate field biomass and crop composition. An accuracy of 79% was reported for seven classes of objects. For an actual dataset of 4400 drone photos, another study [27] developed a new automated weed recognition method that uses convolutional neural networks (CNNs). The suggested CNN LVQ (Learning Vector Quantization) model has an overall accuracy of 99.44% for weed detection. This protects the environment and guarantees agricultural safety while reducing the usage of pesticides. Further research in the literature [28] suggested an efficient weed classification model and an efficient particle swarm optimization (PSO) technique to automatically update and enhance the proposed CNN’s classification accuracy. To categorize the weeds, a total of two datasets were employed, the first including 50 classes of weeds and the second containing 40 classes of weeds. These datasets were also compared with a migration learning model that had already been trained. The two datasets’ prediction accuracy was 98.58% and 97.79%, respectively. Another study [29] employed a deep encoder–decoder CNN with the U-Net architecture to distinguish dirt, weeds, and sugar beet at the pixel level. ResNet50 was used as the encoder block during the training of the U-Net architecture. For this network, the findings revealed an accuracy of 96.06%.

CNN training requires a large set of images. To overcome this problem, previous authors in the literature [30] used migration learning to extract image features for further classification. Three state-of-the-art CNN architectures were tested, and features were selected based on their gain ratios and used as inputs for a support vector machine classifier. The method achieved a hit rate of more than 99%, outperforming nine methods in the literature. The performance of a single learner is usually unsatisfactory due to limited assumption space, falling into local minima, or wrong assumptions of space selection. To address these issues, other research [31] suggests building an ensemble learner by fusing multiple deep CNN learners for lung nodule classification. It was found that the ensemble learner had a higher prediction accuracy compared to the single CNN learner (84.0% vs. 81.7%). The literature [32] proposed an ensemble learning approach to improve the detection of pneumoconiosis in chest X-rays (CXR) using nine machine learning classifiers and multidimensional deep features extracted using the CheXNet-121 architecture. The results show that the integrated ensemble learning exhibits good results (92.68% accuracy, 85.66% Matthews correlation coefficient (MCC), and 0.9302 area under the precision-recall (PR) curve) compared to CheXNet-121 alone and other state-of-the-art techniques. In the task of distinguishing between benign and malignant breast lesions, research [33] compared a support vector machine classifier based on CNN-extracted image features with previously computer-extracted tumor features. Five-fold cross-validation (by lesion) was performed using the area under the receiver operating characteristic curve (ROC) as a performance metric. The results showed that the performance of the classifier based on CNN-extracted features (with migration learning) was comparable to that of the classifier using analytically extracted features (area under the ROC curve (AUC) = 0.81). The literature [34] developed a cascaded machine learning algorithm, which uses a convolutional neural network for images and generates images using a generative adversarial network to train image classifiers. The proposed method outperforms other methods with a sensitivity of 93.33%, specificity of 88.46%, and overall accuracy of 90.24%. We present the above algorithms and their accuracies in tabular form as shown in Table 1.

As can be seen from the above examples, accuracy is one of the criteria for validating a good or bad method, whether it is a feature-based method or a deep learning method. In order to solve the problems of inaccurate recognition results of manually designed features and weak generalization ability of feature extraction, a CNN-SVM model is established. The ‘DeepWeeds’ dataset is used to train the validation test CNN-SVM model, and finally, the test results as well as the confusion matrix are obtained and compared with other algorithms, while the results are also analyzed and discussed. It can be seen that the CNN-SVM weed classification model proposed in this study is effective and could obtain high recognition accuracy, stability, and real-time processing ability.

## 3. Materials and Methods

### 3.1. Dataset

The “DeepWeeds dataset” is a publicly accessible dataset that is utilized in this study [24].

The DeepWeeds dataset contains a total of eight categories of weed images collected from rangeland environments in northern Australia. The images were taken with a high-resolution FLIR Blackfly 23S6C camera (1920 × 1200 pixels) equipped with a machine vision Fujinon CF25HA-1 lens. In this study, 16,800 images are used, of which 7200 are used for validation and testing and 9600 are used for training. The dataset is publicly available through the GitHub repository: https://github.com/AlexOlsen/DeepWeeds (accessed on 15 October 2022).

**Figure 1:**
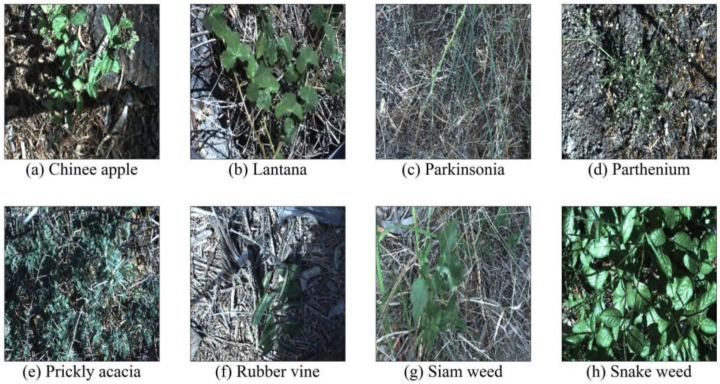
Sample images of each class from the datasets.

The dataset is constructed by combining Chinee apple, Lantana, Parkinsonia, Parthenium, Prickly acacia, Rubber vine, Siam weed, and Snake weed (see Figure 1). An overview of the details relating to the used datasets can be found in Table 2.

### 3.2. Image Pre-Processing

The Facebook Artificial Intelligence Research Institute (FAIR) released PyTorch [35] based on Torch, and we use it in the present work. The data in this study are prepared for training using the Torchvision pre-processing utilities. The data are subjected to several predetermined processes by this function. One of the operations is to resize the images to a uniform size of 224 × 224. To expand the dataset, we randomly rotate the images using the RandomHorizontalFlip(p=0.5), random horizontal flip according to the probability of p, and RandomCrop(32, padding=4) to crop the randomized images. In the paper, the data from the training set are augmented. The original data consisted of 9600 images, and we augmented the data twice (num_augmentations) so that the augmented dataset is twice the size of the original dataset. This paper uses ToTensor() to normalize the images so that the values of the pixels range from 0 to 1 and converts the H ∗ W ∗ C (H: Height, W: Weight, C: Channel) to C ∗ H ∗ W format. Finally, the data are normalized to [−1, 1] by Normalize().

### 3.3. Overview of the Approach

**Figure 2:**
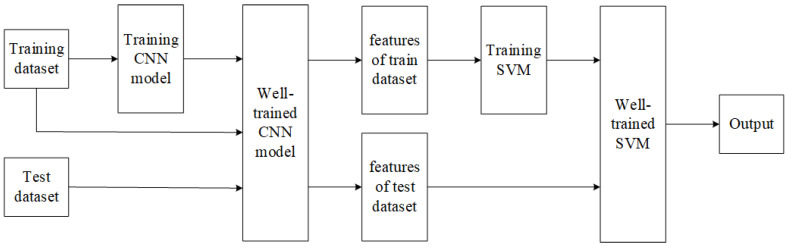
Construction illustration of the CNN-SVM.

The structure of the approach is illustrated in Figure 2. The methodology consists of the following 4 components:

1. Use the training set to train the CNN and obtain the well-trained CNN model.
2. Remove the fully connected layer for feature vector extraction. Extract feature vectors from the training set for training SVM using CNN.
3. The well-trained CNN is again used to extract the feature vectors of the test set.
4. The final extracted feature vectors are classified using the well-trained SVM.

### 3.4. CNN Feature Extraction 

Because of their powerful feature extraction capabilities, CNN networks were utilized to extract features [36]. The original image is input into a CNN network, where the layers are then convolved to generate the image features; the lower layers have a higher level of spatial resolution while the upper layers have a higher level of semantic detail.

In order to evaluate the performance of various CNN features, two CNN networks are selected for comparison. As a machine learning framework, PyTorch is utilized. The chosen CNNs are described below.

**Figure 3:**
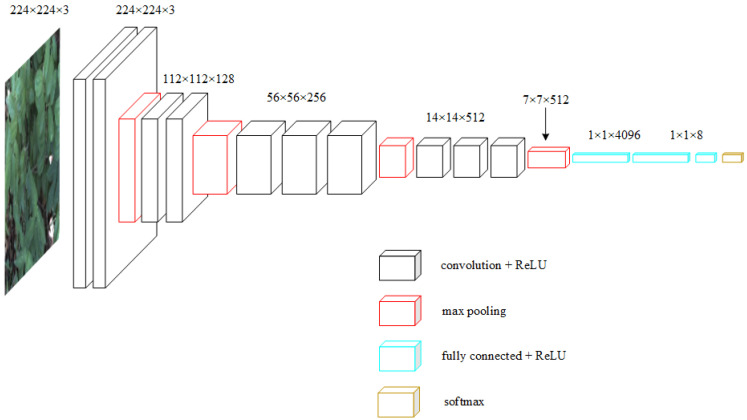
Structural illustrations of the VGG16.

**VGG16** [37] was the winner of the ImageNet image localization competition and runner-up in the classification competition in 2014. In order to reduce the number of parameters while maintaining the number of parameters and the same perceptual field, the VGG16 network employed multiple 3 × 3 convolutional kernels rather than a single large convolutional kernel. It also deepens the network structure and achieves more non-linear operations to enhance the network’s non-linear representation, and finally uses 2 × 2 pooling and removes the normalization layer to further reduce the number of parameters across the board. The VGG16 network benefits from a straightforward structure and strong generalization, and its greater depth yields superior feature extraction outcomes. The structure diagram is shown in Figure 3.

**Figure 4:**
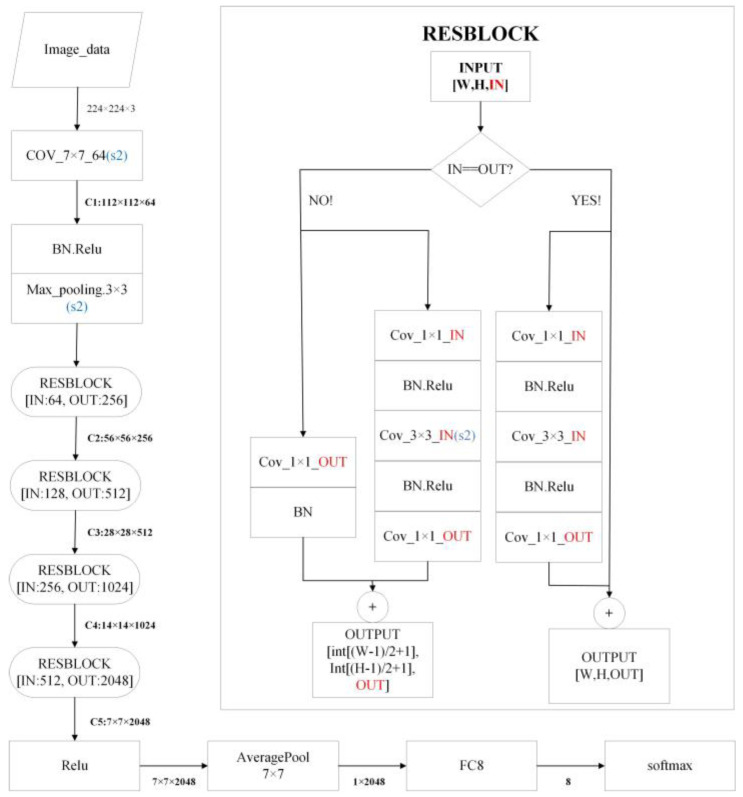
Structural illustrations of the ResNet-50.

**ResNet-50** [38] was first proposed in 2015 due to its “simplicity and practicality” and won first place in the ImageNet competition classification challenge and has since become a popular method for other detection, segmentation, and recognition systems. As the network deepened, there was a drop in the accuracy of the training set, which we can be sure was not due to overfitting (the training set should be highly accurate in the case of overfitting), so the authors proposed a new network for this problem, called Residual Net. ResNet-50 contains two fundamental building blocks: Conv Block and Identify Block. Conv Block provides to vary the network’s dimension because its input and output dimensions are different and cannot be connected in series consecutively. Since they have identical input and output dimensions and may be linked in series, Identity Blocks are used to deepen networks. They are both remnant network architectures. The structure diagram is shown in Figure 4.

### 3.5. SVM Classifier

The SVM is a binary classifier. It is required to create an appropriate multi-class classifier when dealing with multi-class problems because the SVM algorithm is initially developed for binary classification problems. The direct method and the indirect method are two methods for dealing with multi-class classification issues. For this paper, the indirect approach, One-Versus-Rest (OVR SVMs) [39], is selected. 

In this classification method for n categories, only n support vector machines need to be constructed so each support vector machine separates the data of a particular category from the other categories. In testing, the class with the largest output value of the decision function is taken as the class of the test sample. Its $i^{th}$ SVM can be obtained by solving the following optimization problem.

(1) $$\underset{\begin{array}{ccc} \omega^{i} & b^{i} & \xi^{i} \end{array}}{\min}\frac{1}{2}{\left(\omega^{i}\right)}^{T}\omega^{i}+C\sum_{j=1}^{l}\xi_{j}^{i}$$

where ω is a normal vector that determines the direction of the hyperplane and *b* is the displacement term that determines the distance between the hyperplane and the far point. C is the penalty parameter. $\xi_{j}^{i}$ is the hinge loss function. *l* is the training data $\left(x_{1},\;y_{1}\right),\;\ldots ,\;\left(x_{j},\;y_{j}\right)$. The constraints are shown in Equation (2).

(2) $$\begin{array}{c} \{\begin{array}{c} {\left(\omega^{i}\right)}^{T}\phi \left(x_{j}\right)+b^{i}\;\geq \;1-\xi_{j}^{i},\;\;\;\;\;if\;y_{j}=i\;\\ {\left(\omega^{i}\right)}^{T}\phi \left(x_{j}\right)+b^{i}\;\leq -1+\xi_{j}^{i},\;\;\;\;\;if\;y_{j}\;\neq \;i\; \end{array}\\ \xi_{j}^{i}\;\geq 0,\;j=1,\;\ldots ,\;l \end{array}$$

The training data $x_{j}$ are mapped to a high-dimensional space by the ϕ function, $y_{j}\;\in \left\{1,\;\ldots ,\;n\right\}$ is the class of $x_{j}$. Solving the formula yields n decision functions.

(3) $$\begin{array}{c} {\left(\omega^{1}\right)}^{T}\phi \left(x\right)+b^{1}\;,\\ \vdots \\ {\left(\omega^{n}\right)}^{T}\phi \left(x\right)+b^{n}\;. \end{array}\;$$

Then the class to which x belongs is $\arg\underset{i}{\max\;}\left[{\left(\omega^{i}\right)}^{T}\phi \left(x\right)+b^{i}\right]$. 

This paper uses eight different species of weeds, so n=8, *j* = 1,2,…,*l*. Firstly, the first type of weed is taken as the positive sample and the remaining seven as the negative sample, then the second type of weed is taken as the positive sample and the rest as the negative sample, and so on. Finally, eight training sets are obtained and trained separately to obtain eight training result files. When testing, the corresponding test samples are tested with these eight training result files, and the final result obtained is the maximum of these eight results.

## 4. Experimental Setup

### 4.1. Network Training Parameters

All of the experiments in this paper are carried out on a computer with the following specifications: NVIDIA GeForce GTX 1650 SUPER Graphics Processing Unit (GPU), AMD Ryzen 5 5600X 6-Core Processor, 3.70 GHz processing speed, CUDA11.7 parallel computing framework, and cuDNN8.2.0 deep neural network acceleration library. We use Windows 10 Professional Edition as the operating system. With the support of the PyTorch 1.11.0 framework and Python 3.9, deep learning models are created. The following experiments compare different networks fairly by maintaining constants for batch size, learning rate, training epochs, and initial weights. The input size for all networks is 224 × 224 × 3. The optimizers and loss functions used to train the CNN network are *Adam* and *CrossEntropyLoss*. According to Table 3, the network is trained on the DeepWeeds dataset with a learning rate of 0.00005, a batch size of 16, and a training epoch of 450. The initial weight of the network is completely random to further lessen the influence of pre-trained weights. 

### 4.2. SVM Training Parameters

This paper utilized a radial basis kernel function for the SVM classifier of the CNN-SVM model. We adopted the grid search to find out the optimal penalty parameter C and kernel parameter γ by applying the five-fold cross-validation method. We found the optimal values in the range of $C=\left[2^{-3},\;2^{-2},\ldots ,\;2^{3}\right]$ and $\gamma =\left[2^{-5},\;2^{-4},\;\ldots ,\;2^{3}\right]$ for $C=2^{-1}$ and $\gamma =2^{-3}$, respectively, and used them to train the SVM classifier.

### 4.3. ResNet-50-SVM and VGG16-SVM Models

**Figure 5:**
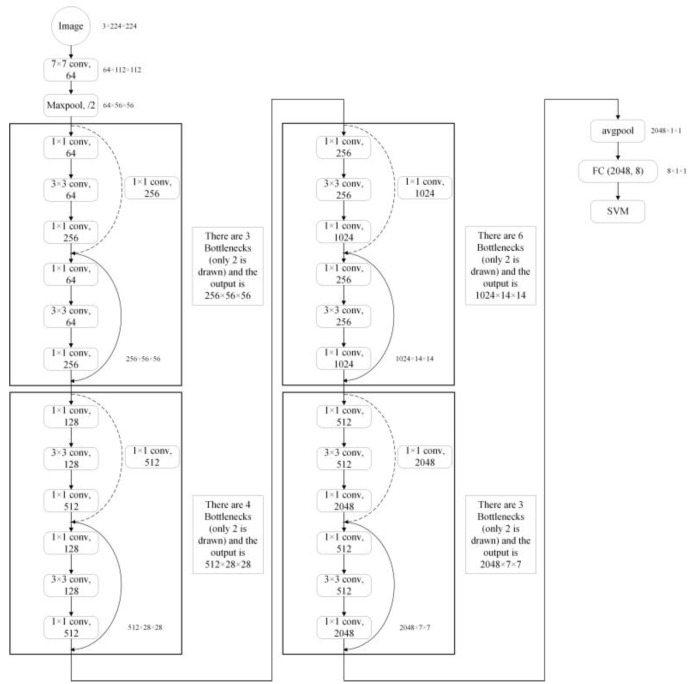
Structural illustration of the ResNet-50-SVM.

**Figure 6:**
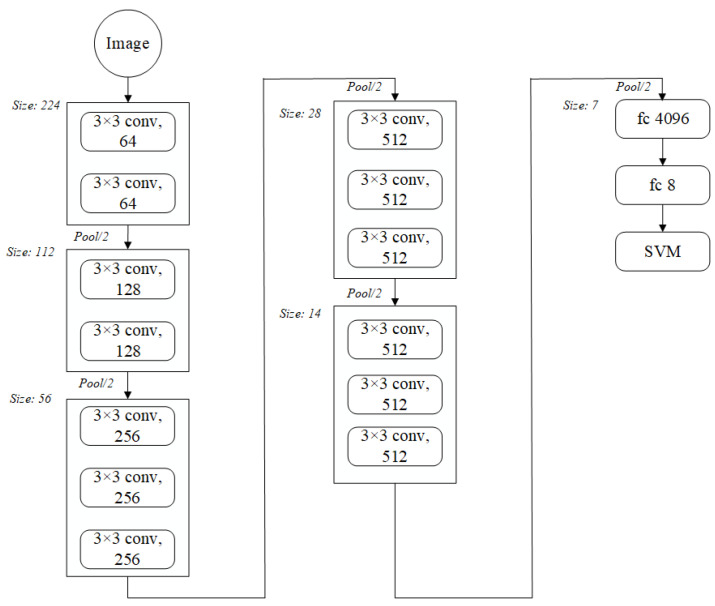
Structural illustration of the VGG16-SVM.

In Section 3, we introduce the structure diagram of the proposed method; here, we present the structural illustrations of ResNet-50-SVM and VGG16-SVM. Figure 5 illustrates the structural illustrations for the ResNet-50-SVM, while the structural illustrations for the VGG16-SVM are presented in Figure 6.

### 4.4. Evaluation of the CNN-SVM Model

Utilizing multiple criteria, including accuracy, precision, recall, and F1 Score, which are described as follows, the networks are tested and assessed, where *tp*, *tn*, *fp*, and *fn* stand for True Positive, True Negative, False Positive, and False Negative, respectively. In this paper, tp means positive samples are identified as positive, tn means negative samples are identified as negative, fp means negative samples are identified as positive, and fn means positive samples are identified as negative. A higher value in each of these indicators indicates better performance.

**Accuracy (Acc):** This is the proportion of the test images whose classes are properly predicted. A greater number indicates a more favorable outcome.

(4) $$accuracy=\frac{tp+tn}{tp+tn+fp+fn}$$

**Precision (P):** This refers to the percentage of samples that the model correctly classified as belonging to a positive class. In general, a model is better the higher its accuracy rate.

(5) $$precision=\frac{tp}{tp+fp}$$

**Recall (R):** Also known as the chase completion rate, the recall rate shows how many of the actual positive samples the classifier can predict.

(6) $$recall=\frac{tp}{tp+fn}\;$$

**F1 Score (F1):** This is described as the average of the recall and accurate rates combined. F1-Score values vary from 0 to 1, with 1 denoting the best and 0 denoting the worst.

(7) $$F1-score=2\times \frac{precision\times recall}{precision+recall}$$

**Confusion Matrix:** For supervised learning in particular, confusion matrices are visualization tools, and they are typically referred to as matching matrices in unsupervised learning. When assessing picture accuracy, it is typically used to compare classification results with actual measured values. The accuracy of the classification results can be displayed in a confusion matrix. The total of each column in the confusion matrix denotes the number of data projected to fall into each category. In the confusion matrix, the anticipated category is displayed in each column. The total quantity of data in each row shows how many occurrences of each type of data there are, and each row’s true attribution category is displayed. The number in each column indicates how many actual data points are expected to belong to that group.

## 5. Experimental Results

**Figure 7:**
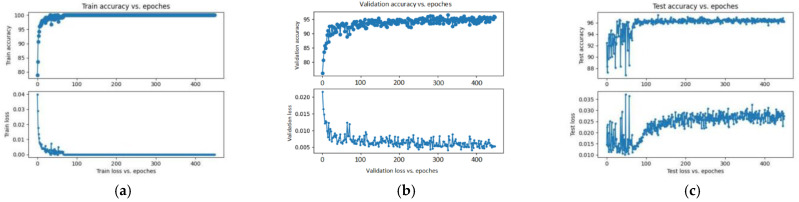
The ResNet-50-SVM training accuracy and loss (**a**); validation accuracy and loss (**b**); testing accuracy and loss (**c**).

Figure 7 shows the training accuracy and training loss of the ResNet-50-SVM and illustrates the testing accuracy and testing loss of the ResNet-50-SVM. It can be seen that the training accuracy is 100% and the training loss is 0. The test accuracy is 97.6%.

**Figure 8:**
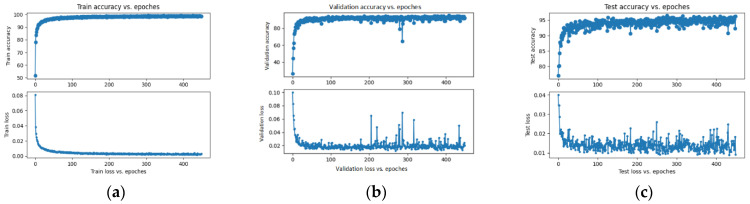
The VGG16-SVM training accuracy and loss (**a**); validation accuracy and loss (**b**); testing accuracy and loss (**c**).

Figure 8 presents the training and testing accuracy and training and testing loss, respectively, for the VGG16-SVM. Training accuracy is 99.6%, training loss is 0.2, and test accuracy is 95.9%.

The recognition accuracy of CNN and CNN-SVM for the DeepWeeds dataset is illustrated in Table 4. This paper tests each network 5 times, obtains their accuracy, and takes the average. The evaluation indicators are shown in Table 5.

A 97.6% accuracy, a 97.7% precision, a 97.6% recall, and an F1 score of 97.6% are obtained by the ResNet-50-SVM. Moreover, 95.9% accuracy, 96.0% precision, 95.8% recall, and 95.9% F1 score are achieved by the VGG16-SVM. 

In comparison, the accuracy of ResNet-50-SVM and VGG16-SVM has been improved by 1.4% and 2.7%, respectively. The result illustrates that the CNN-SVM approach can enhance recognition ability.

**Figure 9:**
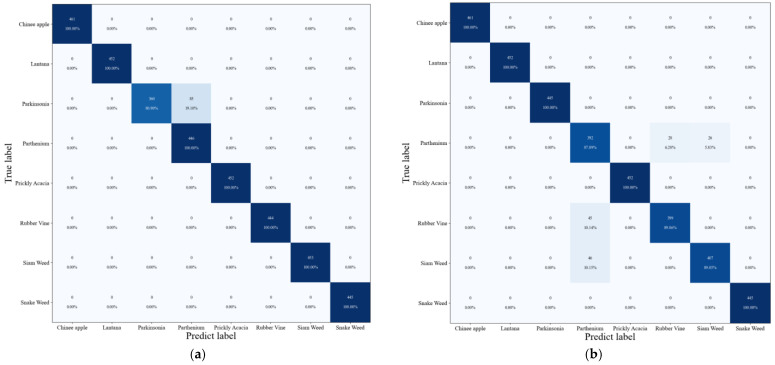
The ResNet-50-SVM confusion matrix for the DeepWeeds dataset (**a**); the VGG16-SVM confusion matrix for the DeepWeeds dataset (**b**).

This paper chose the results of the first experiment to plot the confusion matrix for ResNet-50-SVM (Figure 9a) and VGG16-SVM (Figure 9b).

ResNet-50 has a lower computational complexity but is deeper than VGG16, therefore ResNet-50-SVM is more accurate than VGG16-SVM. In Figure 9a, except for Parkinsonia, the accuracy of all species is 100%. Furthermore, 85 of the 445 test images for Parkinsonia that are used are identified as Parthenium. One of the reasons for classifying Parkinsonia as Parthenium is that the characteristics of the two are relatively similar. In Figure 9b, 446 Parthenium test images are examined, and 28 of them are identified as Siam weed and 26 as Rubber vine. Furthermore, 45 of the 444 test images of Rubber vine are identified as Parthenium and 46 of the 453 test images of Siam weed are identified as Parthenium. The images are difficult to discern because they seem so identical. The VGG16-SVM is unable to distinguish between the classes since the dataset is tiny and the image variations are insufficient. Additionally, there are not enough images collected in the collection. 

**Figure 10:**
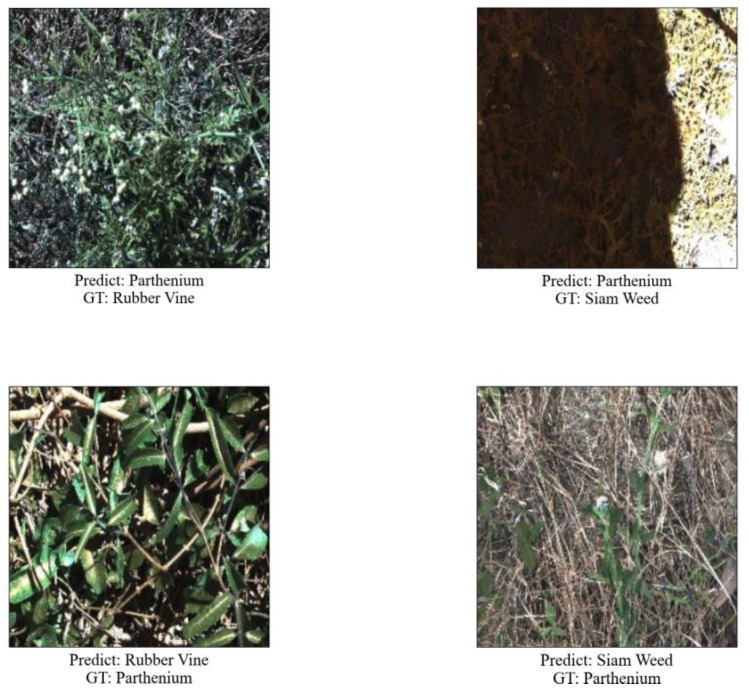
False recognition examples.

Figure 10 also shows some examples of misidentification. Possible reasons for misidentification are that Parthenium, Rubber Vine, and Siam Weed have different backgrounds, similar shapes, and interference due to partial illumination. Considering more differences in the dataset during model training may help improve recognition accuracy.

In conclusion, the advantage of the current work is the high recognition accuracy of CNN-SVM, which is of great help to the subsequent establishment of the image recognition module of the weeding machine. However, the biggest limitation of CNN-SVM is that it is not trained and tested on images under different light conditions, and the recognition accuracy is greatly reduced under sufficient light conditions, and it is not possible to recognize multiple crops and weeds in a single image.

## 6. Discussion

According to the results, ResNet-50-SVM and VGG16-SVM perform better than ResNet-50 and VGG16, which are beneficial for weed recognition. The success of the CNN-SVM-based weed recognition is primarily attributable to different optimization criteria. ResNet-50 and VGG16 used the *softmax* learning algorithm, which is defined as Equation (8).

(8) $$Softmax\left(z_{i}\right)=\frac{e^{z_{i}}}{\sum_{C=1}^{C}e^{z_{i}}}$$

where $z_{i}$ is the output value of the $i^{th}$ node and C is the number of output nodes, i.e., the number of categories for classification. To reduce the training set prediction error, the empirical risk minimization criterion (ERM) is used. ERM makes sure there is a good learning impact when there is a sufficient number of samples. ERM, however, frequently results in overfitting when there is a tiny sample size.

Conversely, structural risk minimization (SRM) [40] is used by the SVM. SRM is a method put forward to avoid overfitting. SRM demands low empirical risk as well as low model complexity, and models with low structural risk typically produce better forecasts for both known and unknowable test data and can bring the expected risk of the SVM over the entire sample set under control. Therefore, the generalization ability of the ResNet-50-SVM and the VGG16-SVM models is more than the ability of the ResNet-50 and VGG16 models.

## 7. Conclusions

In order to solve the problems of inaccurate recognition results of manually designed features and weak generalization ability of feature extraction, a CNN-SVM model is established. The method has excellent generalization performance and achieves stable and high classification accuracy, which can obtain high recognition accuracy, stability, and real-time processing capability, and provides a useful reference for intelligent mechanical weeding. ResNet-50-SVM and VGG16-SVM consist of two parts: CNN feature extraction and SVM classification. First, we train ResNet-50 and VGG16 using the training dataset. Second, in order to train the SVM, features from the training dataset are extracted using the trained CNN networks. The result is finally obtained by classifying the test dataset using the trained SVM. 

We use the public dataset DeepWeeds, which contains a total of eight species of weeds. Using the DeepWeeds dataset, the proposed approaches are also contrasted with state-of-the-art approaches (VGG16, ResNet-50, GoogLeNet, Densenet-121, and PSO-CNN). The state-of-the-art networks with the same dataset are accurate at 93.2%, 96.1%, 93.6%, 94.3%, and 96.9%, respectively. According to the experimental results, the ResNet-50-SVM and VGG16-SVM approaches achieve recognition accuracy rates of 97.6% and 95.9%, respectively. The recognition accuracy of ResNet-50 and VGG16 for the same dataset is 96.1% and 93.2%. It can be seen that the ResNet-50-SVM and VGG16-SVM approaches improve the accuracy of the recognition by 1.5% and 2.7%, respectively. They work well to classify weeds using ResNet-50-SVM and VGG16-SVM, which have great recognition accuracy and real-time processing capabilities. In terms of intelligent agriculture, it is a step toward automatic weeding.

The advantage of the current work is the high recognition accuracy of CNN-SVM, which is of great help to the subsequent establishment of the image recognition module of the weeding machine. 

In the future, we can test the networks in this paper using a multi-species mixed dataset under different lighting conditions. Our ultimate goal is to use the models in this paper for embedded devices (Raspberry Pi, NVIDIA Jetson TX2, etc.) and reduce the cost of automatic weeding.

## Figures and Tables

**Table 1:** Algorithms mentioned in the literature and their accuracy.

Author	Literature Title	Algorithms	Accuracy
Dyrmann, M. [23]	RoboWeedSupport-Detection of weed locations in leaf occluded cereal crops using a fully convolutional neural network	Fully Convolutional Neural Network	86.65%
Olsen, A. [24]	DeepWeeds: A Multiclass Weed Species Image Dataset for Deep Learning	GoogLeNet-based Inception-v3 and ResNet-50	95.1% and 95.7%
Ferreira, A.D. [25]	Weed detection in soybean crops using ConvNets	ConvNets	98%
Haq, M.A. [27]	CNN Based Automated Weed Detection System Using UAV Imagery	CNN LVQ	99.44%
Manikandakumar, M. [28]	Weed Classification Using Particle Swarm Optimization and Deep Learning Models	PSO-CNN	98.58% and 97.79%
Nasiri, A. [29]	Deep learning-based precision agriculture through weed recognition in sugar beet fields	U-Net-CNN	96.06%

**Table 2:** Description of the datasets employed in the experiments.

Species	No. Images
Chinee apple	Train: 1139 Test: 461
Lantana	Train: 1087 Test: 452
Parkinsonia	Train: 1059 Test: 447
Parthenium	Train: 1051 Test: 446
Prickly acacia	Train: 1085 Test: 452
Rubber vine	Train: 1040 Test: 444
Siam weed	Train: 1094 Test: 453
Snake weed	Train: 1045 Test: 445

**Table 3:** Model training parameters.

Parameters	Value	Remarks
Batch size	16	Compute the gradient at a reasonable speed
Training epochs	450	Make certain the network is competent
Learning rate	0.00005	The target function can converge to a local minimum at a reasonable learning rate
Initial weights	Random	No influence of pre-trained weights

**Table 4:** Accuracy performance evaluation of methods (%).

Method	VGG16	ResNet-50	GoogLeNet	Densenet-121	VGG16-SVM	ResNet-50-SVM	PSO-CNN
Test times	Accuracy	Accuracy	Accuracy	Accuracy	Accuracy	Accuracy	Accuracy
1	93.8	96.0	93.2	94.4	96.3	97.8	97.2
2	93.3	96.3	93.5	94.2	96.5	97.7	97.0
3	92.5	95.6	93.4	94.2	95.1	97.5	96.7
4	93.0	96.1	93.9	94.6	95.8	97.4	96.8
5	93.3	96.7	94.0	94.1	95.8	97.6	96.8
Average	93.2	96.1	93.6	94.3	95.9	97.6	96.9

**Table 5:** Comparison of several approaches’ performance on the DeepWeeds dataset (%).

Methods	Accuracy	Precision (Average)	Recall (Average)	F1 Score (Average)
VGG16	93.2	93.2	93.4	93.2
ResNet-50	96.1	96.1	96.2	96.2
GoogLeNet	93.6	93.5	93.6	93.5
Densenet-121	94.3	94.3	94.2	94.2
VGG16-SVM	95.9	96.0	95.8	95.9
ResNet-50-SVM	97.6	97.7	97.6	97.6
PSO-CNN	96.9	96.8	96.9	96.8

## Data Availability

Data are temporarily unavailable due to privacy.
